# Vertical ground reaction forces, paw pressure distribution, and center of pressure during heelwork in working dogs competing in obedience

**DOI:** 10.3389/fvets.2023.1106170

**Published:** 2023-02-10

**Authors:** Danae Charalambous, Christiane Lutonsky, Stefan Keider, Alexander Tichy, Barbara Bockstahler

**Affiliations:** ^1^Department of Companion Animals and Horses, University Clinic for Small Animals, Small Animal Surgery, Section of Physical Therapy, University of Veterinary Medicine, Vienna, Austria; ^2^Department of Biomedical Sciences, Platform of Bioinformatics and Biostatistics, University of Veterinary Medicine, Vienna, Austria

**Keywords:** heelwork, kinetics, obedience, working dogs, ground reaction forces, center of pressure, paw pressure distribution

## Abstract

Heelwork walking is a command that competitive obedience and working dogs are trained to perform. Unlike other canine sports, the research for competitive obedience sport is limited and no research regarding biomechanical gait adaptions during heelwork walking has been published. The aim of the study was to investigate the changes in vertical ground reaction forces, paw pressure distribution (PPD), and center of pressure (COP) of Belgian Malinois during heelwork walking. Ten healthy Belgian Malinois were included in the study. The dogs walked first without heeling (normal walk) and then while heeling on a pressure platform. The comparison between normal and heelwork walking was performed using mixed-effects models. *Post-hoc* analyses were performed using Sidak's alpha correction procedure. During heelwork walking, a significant decrease in the vertical impulse and stance phase duration (SPD) and a significant increase in the craniocaudal index and speed of COP was observed in the forelimbs compared to normal walking. At the hindlimbs, a significant increase in vertical impulse and SPD was observed during heelwork walking. Regarding PPD, a significant decrease of vertical impulse was observed at the cranial quadrants of the right forelimb and craniolateral quadrant of the left forelimb during heelwork. The area was significantly decreased at the craniolateral quadrant of the left forelimb and the time for the peak vertical force was prolonged significantly at the caudal quadrants of the right forelimb during heelwork walking. The vertical impulse was significantly increased in all quadrants of the hindlimbs except the craniolateral quadrant of the left hindlimb. The effect of these changes on the musculoskeletal system of working dogs should be investigated in further studies, using electromyography and kinematic analysis.

## 1. Introduction

Police, military, search and rescue, and security dogs are some examples of working dogs, which, alongside with the handler, provide vital support to the society (1). The financial investment to acquire, train, and maintain working police and military dogs makes them very valuable. Understanding causes of injury can provide optimal care and preventive measures that could prolong the working life of a service dog and improve animal welfare (2, 3). Police and military dogs are at an increased risk of orthopedic disease compared to dogs living as pets (2, 4, 5). In a study investigating the causes of loss from active duty among German Shepherd police dogs, the single most important cause was the inability to cope with the physical demands of the job (61/94 dogs or 65%). The median age of retirement was 6.6 years of age and only 40% reached the desired age of retirement of 8 years, most commonly due to orthopedic diseases (3). Given the high prevalence of musculoskeletal diseases, such as hip dysplasia, lumbosacral stenosis (6), semitendinosus myopathy (7–10) and supraspinatus tendinopathy (11) in working dogs investigations should be conducted to determine how each training affects the animal's body.

Certain areas of working dog training paved the way for dog sports. Competitive obedience was first introduced in 1930 as an adaptation of the military dog's work. Originally, the purpose of this competition was to show that the dog can work with their owner and follow specific commands which ensure a good compliance in daily life (11). Over the years, competitive obedience has evolved and more commands have been included in the competition trials. According to the Federation Cynologique Internationale (FCI), three main classes exist for competitive obedience (12).

**Figure 1:**
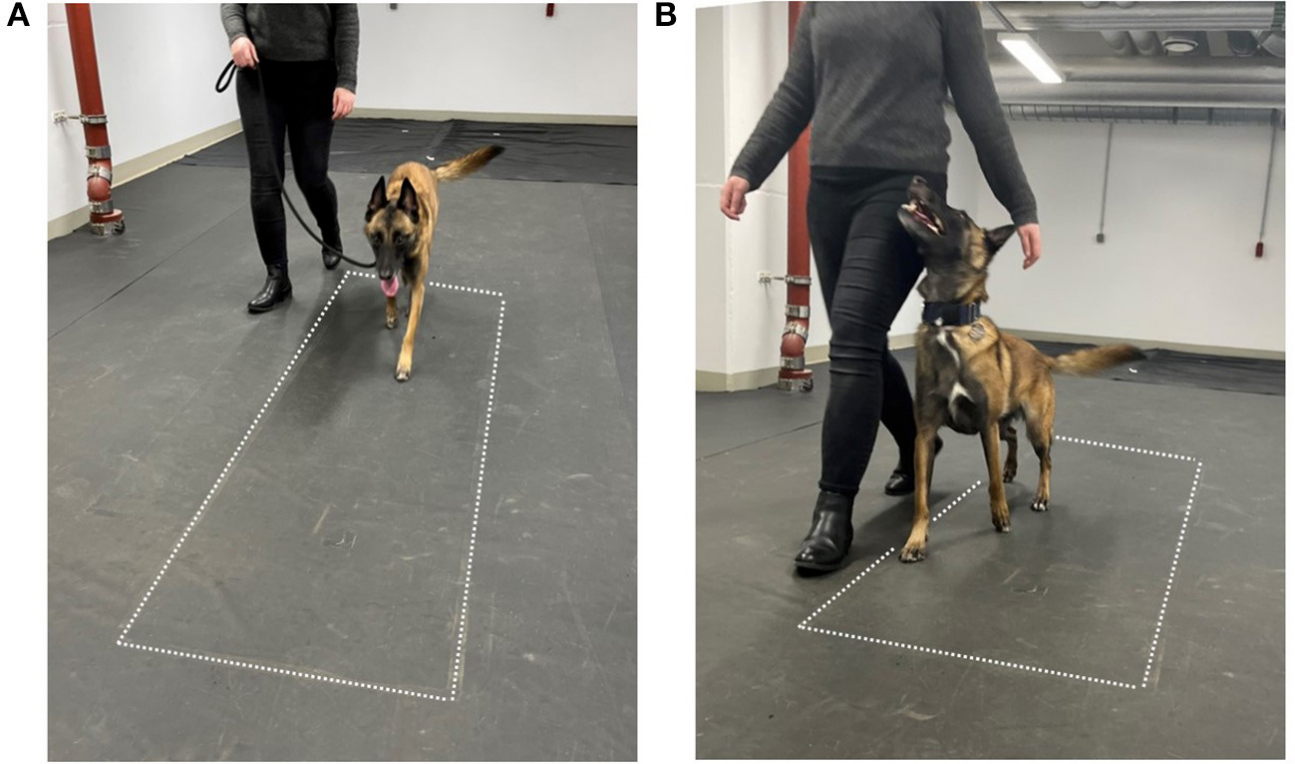
Measurement setup. **(A)** Normal walking, **(B)** heel work position. The dotted lines indicate the position of the pressure measurement plate.

Heelwork is a basic command and is included in all levels. This should not be confused with normal walking at the handler's side, where the dog usually faces forward (Figure 1A). Dogs are trained to walk close to the handler's left leg, with their head looking up and to the right toward the handler (Figure 1B) while performing a series of commands (11). The guidelines regarding heelwork include the following: the dog's shoulder should be approximately level with and reasonably close to the handler's leg at all times (13) and that the dog's head position should in no way compromise its top line or impair the natural movement of the dog. Moreover, the FCI states that “the dog should move naturally but what is seen as a natural neck and backline depends also on the breed” (12).

The heelwork position observed in obedience sports and other working dogs differs greatly from the physiological posture observed during normal walking (14)—due to the lifting of the head and its rotation to the right. Although research in this specific sport is limited, it has been proposed in textbooks that competitive obedience dogs experience chronic strain injuries to the shoulders, such as supraspinatus tendinopathy, especially at the left shoulder due to heeling (11).

Due to the increased awareness of preventive medicine, the specific posture that these dogs adapt has attracted research interest. The apparent neck angle of competitive obedience dogs shows a wide variation from 97.2° (±2.0) to 169.7° (±2.8) (mean 123.4°). However, there is no significant correlation between the apparent neck angle and the judges' scores, nor between the duration and neck angle. This suggests that it does not create an advantageous speed when working in heelwork position (14). While further research investigated the human preferences for heelwork positions during competitive obedience in the United Kingdom (15), the effect of the changed head position on the animals body has not been evaluated using kinetic and kinematic analysis nor electromyography.

The evaluation of the ground reaction forces (GRFs) has gained interest in the last decade due to the non-invasive and relatively easy to perform measurements. These are used to assess normal motion in healthy individuals (16–19) to evaluate efficacy of treatments (20–23) and as an objective diagnostic tool (24, 25). Evaluation of the GRFs provides data on the forces generated between the limb and the ground during the stance phase. Numerous values can be obtained during a gait analysis, including PFz and IFz for each limb. Moreover, each paw can be further divided in quadrants (cranio-medial, cranio-lateral, caudo-medial, and caudo-lateral) to examine the distribution of the pressure within the paw (PPD, paw pressure distribution). Important findings include the normal forces exerted on each digit or pad during walking and other activities in sound animals (24, 25) and changes in the affected and other limbs in orthopedically diseased animals (26, 27).

In recent years, there has been additional interest in veterinary research in the center of pressure (COP) which has been studied in humans for decades. The COP is the instantaneous vector where the GRFs act and reflects the trajectory of the center of mass (COM) of the body. During ground contact, the position of the COP changes continuously, thus creating the COP path. Displacements of the COM lead to a displacement of the COP, which can be easily measured by for example force plates or pressure plates. In human medicine, the COP is used as an indirect way to measure the postural sway (28). A variety of COP are used in research, which can be derived from the measured COP path. Typical examples are (a) the COP-area (also called statokinesiogram), determined by the recorded points of the COP trajectory; (b) the mean speed of the movement of the COP (COP-speed, mm/s); (c) the mean distance of all COP points to the center point of all COP points (COP-radius); (d) the COP excursion index and the craniocaudal index, quantifying the mediolateral and craniocaudal displacement of the COP respectively (29–33).

It should be noted that both the body COP (e.g., in humans between the legs) and the COP under the legs (limb COP) can be evaluated as well during standing (static posturography) as well during motion (dynamic posturography). If the COP is evaluated during standing it is generally assumed, that lower values of the mentioned values are indicative for better stability (28, 29, 33–39). In humans such measurements are widely used in different conditions. As a few examples: it was shown that in patients with knee osteoarthritis COP-parameters correlated with the severity of the osteoarthritis (40) and in such with hip osteoarthritis significant differences occurred in numerous COP parameters (41). Further, there is a wide body of evidence that impairment of postural stability is associated with aging and Alzheimer's Disease (42, 43). In veterinary medicine, newborn foals showed a higher amplitude and velocity of COP movements compared data collected during the following months, which was interpreted as an increased stability during the growth (44) and senior dogs showed a higher COP displacement compared to younger dogs (39). Also lame dogs with elbow osteoarthrosis showed a significant increase in COP-area and greater and asymmetric oscillations in mediolateral and craniocaudal direction (33). Similarly, lame horses showed a significantly higher mediolateral COP displacement (30).

When the COP measured in motion, as recently done in veterinary medicine, it can also be used to describe the foot dynamics and lame dogs show significant differences in COP parameters compared to sound dogs. A significant increase in COP-area was found in lame dogs in the in the affected limb (31, 32) as well as in the hindlimbs in dogs with elbow osteoarthrosis (31), indicating an increased instability (32). Furthermore, affected dogs show a significant increase in the caudal margin (the distance between the most caudal point of the paw and the most caudal point of the COP path) and a significant decrease in craniocaudal excursion index and mediolateral COP excursion (31, 32), while the latter has been interpreted as a sign for an increased pad deformation (32). A lateral migration of the COP, which was also found in lame dogs with elbow osteoarthrosis, was interpreted as a sign of joint instability and body posture modification (38). Similar findings, in the sense of an increases mediolateral displacement of the COP was found in dogs with coxarthrosis (31).

Further also connections can be made between the PPD and the COP. In human medical research, the interaction between the size of the foot contact area and the plantar pressure distribution and stability has been investigated using the COP excursion (45–47), where again a larger COP excursion was interpreted as a sign of reduced stability. It has been proposed that even little changes in the support surface result in instability due to a reduction in sensory information (45). In veterinary medicine, research has not focused on the influence of PCA nor PPD on stability, and therefore COP parameters yet.

In summary, GRF, COM, and COP are closely related and can be used to describe the effects of various factors, such as aging, orthopedic and neurological diseases.

Due to the specific body position during heelwork, it can be assumed that heelwork leads to changes in the above-mentioned parameters. While there is no information on the influence of the head- and neck position on vertical GRF (vGRF) in dogs, research in equines found an effect of the head position on the vertical impulse (IFz), stride length, and stance duration of the forelimbs. A higher degree of collection of the head and neck in horses results in a pronounced shift in impulse toward the hindlimbs [without a similar increase of the peak vertical force (PFz)] (48). However, veterinary medical research does not provide information regarding the effect on COP parameters.

The aim of the present study was to investigate the changes in vGRFs, COP, and PPD of Belgian Malinois participating in competitive obedience during heelwork walking. First aim was to measure the GRF, assuming that lifting the head leads to increased forces in both hindlimbs and that turning the head to the right additionally leads to an increased force in the left forelimb, resulting in an asymmetrical loading on the contralateral limb pair as shown by the SI. Second aim was to investigate whether the altered vGRF also lead to changes in PPD, or whether the loading on the paw quadrants is unchanged. Since COP is affected by changes in PPD as described in human medicine (47), the 3rd aim was to investigate whether altered PPD is also reflected in changes in COP parameters.

## 2. Material and methods

### 2.1. Ethics

The study was approved by the Ethics and Animal Welfare Committee of the University of Veterinary Medicine, Vienna, in accordance with the university's guidelines for “Good Scientific Practice” (Ref. No. 18/03/97/2014 and ETK-133/08/2022).

### 2.2. Animals and inclusion criteria

Based on a preliminary master thesis (49), sample size was determined using GPower (Version 3.1.9.6, University Kiel, Germany). The IFz (normalized to total force) of the values of Malinois collected in this study during normal walking and heelwork were used as baseline data. The IFz showed significantly higher values in the hind legs during heelwork (HL 22.35 ± 3.10%, HR 22.98 ± 2.94%) than during normal walking (HL 19.46 ± 1.02% *p* = 0.03, HR 19.56 ± 1.58% *p* = 0.02). With α error probability 0.05 and a power of 0.90 we calculated based on that a sample size of *n* = 10 for HL and *n* = 7 for HR.

Twelve to ten-year-old Belgian Malinois were included in the study. The animals should present with normal orthopedic and neurological examination results to be included. The orthopedic examination included visual assessment of lameness and palpation of joints. Furthermore, an SI < 3% was required, as an SI of up to 3% is considered normal for dogs and values higher than 3% are interpreted as lameness (26, 27, 31).

Twelve Belgian Malinois dogs were examined for the study. Two dogs were excluded, one due to pain at the palpation of the hip joint and one dog due to a SI of 3.08%. Seven females and three males were evaluated for the present study. The mean age was 4.67 ± 1.26 years (median = 4.95 years, minimum = 3 years, maximum = 7 years) and the mean body mass was 27.46 ± 6.58 kg (median = 26 kg, minimum = 20.6 kg, maximum = 41 kg).

### 2.3. Equipment

A 203- × 54.2-cm pressure measurement plate [FDM Type 2 from Zebris Medical GmbH, Allgäu, Germany (27, 31, 50–55)] was used, which measures the pressure of the dog's paws through 15,360 piezoelectric sensors and a sampling rate of 100 Hz. The plate was covered with a black, 1-mm-thick rubber mat composed of polyvinyl chloride to avoid slipping. To assign the measured values to the correct limb of the respective test during data evaluation, each measurement run was filmed with a Panasonic camera, model NV-MX500.

### 2.4. Measurement procedure

Same-day measurements were acquired for each dog. The dogs were first allowed to become accustomed to the examination room and the people involved. For this purpose, they were allowed to move freely in the room. As soon as the animal became accustomed to the environment, they were subjected to a standard analysis of the vGRFs during a normal walk, as described by Reicher et al. (31), including an afterward analysis of the vGRFs while heeling. The animals were on the left side of the owner, which is the side that they usually walk next to the owner. This was repeated until a minimum of four valid steps had been collected for each limb and gait (normal walk and heelwork walk). Valid passes during normal walk included those where the dog had crossed the plate without changing pace, turning its head, pulling on the lead, and touching the owner. During heelwork walk, valid passes were those in which the dog performed the command without interruption. Moreover, if the dog had body contact with the handler, those measurements were excluded for both gaits. Heelwork was achieved in the same way as in obedience competitions. The difference in speed at which the dogs crossed the plate had to be within a range of ±0.3 m/s and an acceleration of ±0.5 m/s^2^ (56, 57). The values obtained during a normal walk were compared with the values obtained during a heelwork walk.

### 2.5. Data analysis

The data were analyzed with the custom software Pressure Analyzer (Michael Schwanda, version 4.6.5.0) and then exported to Microsoft^®^ Excel^®^ 2016. The individual pawprints recorded during the valid passes were manually assigned to the corresponding limb with the help of the recorded video. The standard method of assessment includes the identification and assignment of each limb as left forelimb (FL), right forelimb (FR), left hindlimb (HL), and right hindlimb (HR).

### 2.6. Parameters under investigation

The following parameters were used for the evaluation of the vGRFs and temporo-spatial parameters:

- Mean speed (m/s) and acceleration (m/s^2^) which were calculated based on the left forelimb;
- Peak vertical force (PFz in N);
- Vertical impulse (IFz in N/s);
- The PFz and the IFz were normalized with the following formula and expressed as a percentage of total forces:
  $$\begin{array}{l} Value\;in\;\%\;of\;total\;force\\ \;=\frac{XFzLx\;}{\left(XFzFL+XFzFR+XFzHL+XFzHR\right)}*100 \end{array}$$
  Where: XFz is the mean value of PFz or IFz of the valid steps, Lx is the limb under investigation, FL is the left forelimb, FR is the right forelimb, HL is the left hindlimb, and HR is the right hindlimb.
- SI expressed as a percentage (SI%) was calculated for both parameters (PFz and IFz) according to the following equation:
  $$SIXFz\;(\%)=abs\left(\frac{\left(XFzLLx-XFzRLx\right)}{\left(XFzLLx+XFzRLx\right)}\right)*100$$
  Where XFz is the mean value of PFz or IFz of the valid steps, LLx is the left fore- or hindlimb, and RLx is the right fore–hindlimb; perfect symmetry between the right and left fore- or hindlimbs was assigned a value of 0%.
- Mean stance phase duration (SPD) in seconds.
  The mean duration of the stance phase was normalized with the following formula, expressed as the percentage of the total SPD (SPD%).
  $$\begin{array}{l} SPD\;\left(\%\right)\\ \;=\frac{XmDStPhLx\;}{\left(XmDStPhFL+XmDStPhFR+XmDStPhHL+XmDStPhHR\right)}\\ \;*100 \end{array}$$
  Where XmDStPh is the value of the mean duration of the stance phase of the valid steps, Lx is the limb under investigation, FL is the left forelimb, FR is the right forelimb, HL is the left hindlimb, and HR is the right hindlimb.
- Time of occurrence of PFz as a percentage of the stance phase of the respective limb (TPFz%).
- Paw contact area (PCA) of each limb as a percentage of the contact area (cm^2^).
- Step length (SL) of each limb in meters.
- Reach which is the distance between the center of the forelimb and the center of the hindlimb on the same side in the direction of movement in cm.

The evaluation of the vertical force distribution in the paws was performed according to Moreira et al. (26) as follows.

Each paw was equally divided into four quadrants, craniomedial (CrM), craniolateral (CrL), caudomedial (CdM), and caudolateral (CdL). This was achieved by calculating the midpoint of the maximum length in the cranial/caudal and medial/lateral directions of each paw by the software. The parameters under investigation were the PFz, IFz, TPFz, and area. The PFz and IFz were normalized to the total force and presented as % (i.e., PFz% and IFz%), in which the sum of PFz% and IFz% of the 16 quadrants was equal to 100%.

The formula used for the above values was:


$$\text{TFnk}\left(\%\right)=\frac{100*X_{nk}}{\sum_{k=1}^{4}\sum_{n=1}^{4}X_{nk}}$$

Where *X* represents PFz% or IFz%, *n* represents a limb (FL, FR, HL, and HR), and *k* represents one quadrant (CrL, CrM, CdL, and CdM).

The TPFz was normalized to the duration of the stance phase and the area was normalized to the contact area.

For the statistical analysis, each quadrant was compared for the two walks (normal and heelwork walk).

The evaluation of the COP was conducted according to Reicher et al. (31) and Lopez et al. (32) as follows:

- COP-area: The COP-area is a measurement of the area covered by the COP movement. It was normalized to the PCA and expressed as a percentage (COP-area%).
- COP-speed: The COP-speed is the mean speed of the movement of the COP (COP-Sp, mm/s).
- COP-radius: The COP-radius is the mean distance of all COP points to the center point of all COP points. This parameter was also normalized to the PCA and given as a percentage (COP-radius%).
- Caudal margin (mm): Caudal margin is the distance between the most caudal point of the paw and the most caudal point of the COP path.
- Craniocaudal index (%): Craniocaudal index is the COP length (the distance between the first and the last COP point in the craniocaudal axis) in relation to the paw length. It was calculated with the following formula:
  $$Craniocaudal\;index\;\left(\%\right)=\frac{COP\;length}{paw\;length}*100$$
- COP excursion index (%): COP excursion index is the lateromedial excursion (the distance between the first and the last COP point in the mediolateral axis) of the COP related to the paw width. It was calculated with the following formula:
  $$COP\;excursion\;index\;\left(\%\right)=\frac{lateromedial\;excursion}{paw\;width}*100$$

### 2.7. Statistical analysis

All statistical analyses were performed using IBM SPSS, version 28. The difference between normal walk and heelwork in the investigated parameters was evaluated using linear mixed-effects models, where the condition (normal walk vs. heelwork) was added to the model as a fixed within subjects effect *Post-hoc* analyses were performed using Sidak's alpha correction procedure. A *p*-value below 5% (*p* < 0.05) was seen as significant.

## 3. Results

### 3.1. Speed and acceleration

No significant differences were found for the speed and acceleration between normal walk (1.34 ± 0.21 m/s, −0.04 ± 0.10 m/s^2^) and heelwork (1.41 ± 0.24 m/s, 0.01 ± 0.09 m/s^2^) (Table 1) and they were within the defined ranges (Chapter 2.4).

**Table 1:** Mean values, standard deviation, and *p*-values of the speed and acceleration based on the left forelimb between normal walking and heelwork walking.

	**Speed**			**Acceleration**		
	**Mean** ±**SD**		***p*** **-value**	**Mean** ±**SD**		***p*** **-value**
	**Normal walk**	**Heelwork walk**	**Normal walk**	**Heelwork walk**	**Normal walk**	
FL	1.34 ± 0.21	1.41 ± 0.24	0.495	−0.04 ± 0.10	0.01 ± 0.09	0.218

FL, left forelimb; mean ± SD, mean and standard deviation.

### 3.2. Vertical ground reaction forces and temporo-spatial parameters

No significant differences were found between heelwork and normal walk regarding PFz, TPFz, reach, SL, and PCA (Table 2).

**Table 2:** Mean values, standard deviation, and significant differences of the ground reaction forces and temporo-spatial parameters for each limb between normal walking and heelwork walking.

	**PFz (%)**			**IFz (%)**			**TPFz (%)**			**SPD (%)**		
	**Mean** ±**SD**		***p*** **-value**	**Mean** ±**SD**		***p*** **-value**	**Mean** ±**SD**		***p*** **-value**	**Mean** ±**SD**		***p*** **-value**
	**Normal walk**	**Heelwork walk**		**Normal walk**	**Heelwork walk**		**Normal walk**	**Heelwork walk**		**Normal walk**	**Heelwork walk**	
FL	29.18 ± 1.40	29.57 ± 1.69	0.582	30.59 ± 0.92	26.76 ± 2.69	0.001^*^	36.17 ± 8.17	40.82 ± 7.12	0.192	25.90 ± 0.65	22.88 ± 2.39	0.003^*^
FR	29.51 ± 1.29	30.37 ± 2.51	0.351	31.16 ± 1.04	26.47 ± 3.44	0.002^*^	37.73 ± 10.02	41.11 ± 8.16	0.418	25.85 ± 0.61	22.23 ± 2.39	0.001^*^
HL	20.86 ± 1.13	19.86 ± 1.41	0.095	19.27 ± 0.98	23.20 ± 2.46	0.001^*^	31.22 ± 11.26	31.02 ± 7.76	0.965	24.22 ± 0.70	27.11 ± 1.88	0.001^*^
HR	20.45 ± 1.50	20.20 ± 2.34	0.785	18.97 ± 0.98	23.57 ± 3.49	0.002^*^	30.34 ± 10.78	30.28 ± 7.13	0.989	24.03 ± 0.54	27.79 ± 2.77	0.003^*^
	**PCA (%)**			**SL (m)**			**Reach (cm)**					
	**Mean** ±**SD**		***p*** **-value**	**Mean** ±**SD**		***p*** **-value**	**Mean** ±**SD**		***p*** **-value**			
	**Normal walk**	**Heelwork walk**		**Normal walk**	**Heelwork walk**		**Normal walk**	**Heelwork walk**				
FL	26.66 ± 0.46	26.44 ± 0.98	0.540	0.88 ± 0.10	0.79 ± 0.17	0.159						
FR	26.94 ± 0.37	26.43 ± 1.39	0.287	0.89 ± 0.11	0.81 ± 0.15	0.198						
HL	23.24 ± 0.47	23.41 ± 1.10	0.664	0.89 ± 0.11	0.88 ± 0.12	0.768	6.48 ± 5.16	3.31 ± 6.43	0.241			
HR	23.15 ± 0.43	23.70 ± 1.28	0.222	0.89 ± 0.11	0.90 ± 0.14	0.803	4.49 ± 8.93	3.02 ± 6.36	0.676			

FL, left forelimb; FR, right forelimb; HL, left hindlimb; HR, right hindlimb; mean ± SD, mean and standard deviation; PFz%, peak vertical force as a percentage of total force; IFz%, vertical impulse as a percentage of total force; TPFz%, time to PFz as a percentage of stance phase duration; SPD%, stance phase duration as a percentage of total stance phase duration; PCA%, paw contact area; SL(m), step length. Significant differences within the groups are marked with *.

**Figure 2:**
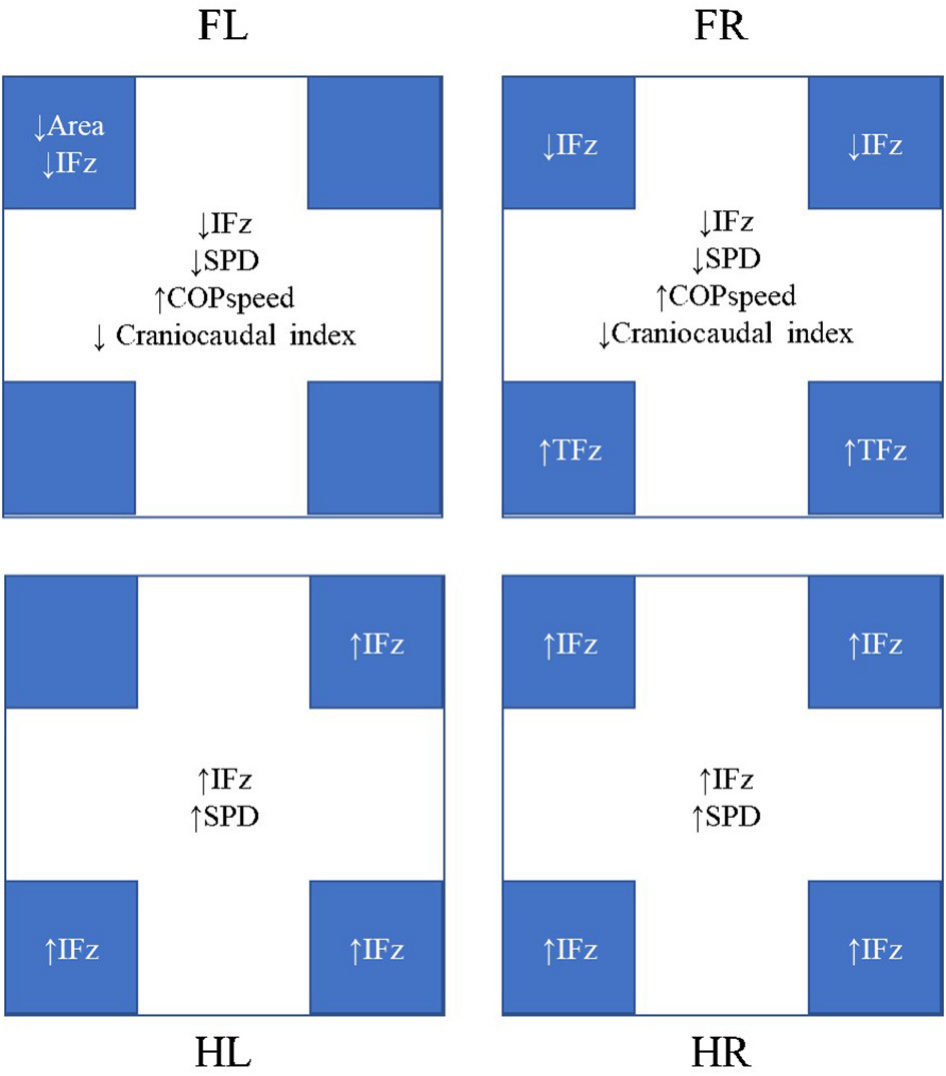
Significant differences were observed during heelwork walking compared to normal walking. The results in the center of each box represent the changes in each limb, whereas the results in the blue boxes represent the changes in the quadrants. FL, left forelimb; FR, right forelimb; HL, left hindlimb; HR, right hindlimb; IFz, vertical impulse; SPD, stance phase duration; TPFz, time to peak vertical force. All mean values, standard deviations, and *p*-values are given in Tables 2, 4, 6, 7.

During heelwork, eight dogs increased their SI of PFz where 7/10 dogs reached an SI of PFz >3% (range 3.18–6.47%), with five dogs displaying a higher PFz in the FR, and two on the FL (Table 3). Although this resulted in comparable mean values for FL and FR PFz, without a significant difference to a normal walk, a significantly higher SI of PFz (3.56%) during heelwork than in the normal walk (1.15%, *p* = 0.012, Table 4) was observed. In the hindlimbs (Table 3), six dogs displayed a higher SI of PFz than in normal walk, but only four dogs had an SI of PFz >3% (range 3.68–7.05%) (two dogs with higher PFz values on the HL and two on the HR). Neither PFz nor SI of PFz showed a significant difference in comparing a normal walk and heelwork (Tables 2, 4). Significant differences between heelwork and normal walk are displayed in Figure 2.

**Table 3:** Mean values of PFz and symmetry index of PFz of the forelimbs and hindlimbs during normal walk and heelwork walk for each dog and all dogs.

**Dog**	**Normal walk**			**Heelwork walk**			**Normal walk**			**Heelwork walk**		
	**PFz%**		**SI% of PFz**	**PFz%**		**SI% of PFz**	**PFz%**		**SI% of PFz**	**PFz%**		**SI% of PFz**
	**FL**	**FR**	**Forelimbs**	**FL**	**FR**	**Forelimbs**	**HL**	**HR**	**Hindlimbs**	**HL**	**HR**	**Hindlimbs**
1	29.79	30.46	1.11	29.06	30.97	3.18	19.87	19.89	0.05	20.72	19.25	3.68
2	31.28	30.69	0.95	31.82	29.17	4.34	19.54	18.49	2.76	19.4	19.61	0.54
3	27.58	29.22	2.89	31.93	32.19	0.41	21.91	21.29	1.44	18.26	17.62	1.78
4	30.01	30.46	0.74	28.77	32.75	6.47	19.93	19.6	0.83	20.09	18.39	4.42
5	29.87	28.97	1.53	30.8	32.85	3.22	21.17	19.99	2.87	18.48	17.87	1.68
6	28.29	29.2	1.58	28.7	28.42	0.49	21.19	21.31	0.28	20.98	21.91	2.17
7	28.92	29.98	1.80	27.7	30.71	5.15	21.04	20.06	2.38	20.69	20.9	0.50
8	30.8	30.98	0.29	31.17	30.86	0.50	19.36	18.86	1.31	17.67	20.3	6.93
9	26.99	26.77	0.41	27.72	24.46	6.25	22.6	23.64	2.25	22.22	25.59	7.05
10	28.26	28.34	0.14	28.01	31.33	5.59	22.04	21.36	1.57	20.05	20.61	1.38
Mean ± SD	29.18 ± 1.40	29.51 ± 1.29	1.15 ± 0.84	29.57 ± 1.69	30.37 ± 2.51	3.56 ± 2.40	20.86 ± 1.13	20.45 ± 1.50	1.58 ± 0.99	19.86 ± 1.41	20.20 ± 2.34	3.01 ± 2.43

Black boxes indicate which forelimb or hindlimb has higher values. Green indicates decrease and red indicates increase of the values during heelwork walk compared to normal walk. Orange indicates increase of the values during heelwork walk compared to normal walk but not above 3% (normal).

FL, left forelimb; FR, right forelimb; HL, left hindlimb; HR, right hindlimb; PFz, peak vertical force; SI%, symmetry index; SD, standard deviation.

**Table 4:** Mean values, standard deviation, and significant differences of the symmetry index for the peak vertical force and vertical impulse between normal walking and heelwork walking.

	**Symmetry PFz (%)**			**Symmetry IFz (%)**		
	**Mean** ±**SD**		***p*** **-value**	**Mean** ±**SD**		***p*** **-value**
	**Normal walk**	**Heelwork walk**		**Normal walk**	**Heelwork walk**	
Forelimbs	1.15 ± 0.84	3.56 ± 2.40	0.012^*^	1.25 ± 0.61	3.62 ± 2.89	0.030^*^
Hindlimbs	1.58 ± 0.99	3.01 ± 2.43	0.110	1.62 ± 0.81	3.61 ± 2.36	0.028^*^

Mean ± SD, mean and standard deviation; PFz%, peak vertical force as a percentage of total force; IFz%, vertical impulse as a percentage of total force. Significant differences within the groups are marked with ^*^.

IFz decreased significantly at the forelimbs (FL: *p* = 0.001, FR: *p* = 0.002). The SI of IFz of the forelimbs (Table 4) during heelwork was increased in eight dogs, with five dogs displaying values >3% (range 3.11–9.9%); here, three dogs had higher IFz on the FL (Table 5). The SI of IFz of the forelimbs during heelwork (3.62%) was significantly increased compared to a normal walk (1.25%, *p* = 0.030). IFz increased in both hindlimbs (HL: *p* = 0.001, HR: *p* = 0.002). Seven dogs increased their SI of IFz, with six dogs displaying an SI of IFz > 3% (range 3.19–6.45%), with four of them displaying higher values in the HR and two in the HL (Table 5). The SI of IFz of the forelimbs and hindlimbs during heelwork (3.61%) was significantly increased compared to a normal walk (1.62%, *p* = 0.028, Table 4).

**Table 5:** Mean values of IFz and symmetry index of IFz of the forelimbs and hindlimbs during normal walk and heelwork walk for each dog and all dogs.

**Dog**	**Normal walk**			**Heelwork walk**			**Normal walk**			**Heelwork walk**		
	**IFz%**		**SI% of IFz**	**IFz%**		**SI% of IFz**	**IFz%**		**SI% of IFz**	**IFz%**		**SI% of IFz**
	**FL**	**FR**	**Forelimbs**	**FL**	**FR**	**Forelimbs**	**HL**	**HR**	**Hindlimbs**	**HL**	**HR**	**Hindlimbs**
1	30.55	32.01	2.33	27.2	31.2	6.85	19.21	18.23	2.62	22.04	19.56	5.96
2	30.58	31.46	1.42	23.77	22.03	3.82	19.13	18.83	0.79	27.38	26.82	1.03
3	30.8	32.05	1.99	28.12	27.46	1.19	18.95	18.21	1.99	22.48	21.95	1.19
4	31.92	32.39	0.73	29.8	27.23	4.51	17.59	18.1	1.43	22.72	20.26	5.72
5	31.15	31.92	1.22	29.26	30.77	2.52	18.93	17.99	2.55	19.8	20.18	0.95
6	30.67	31.37	1.13	24.79	25.84	2.08	19.27	18.69	1.53	25.01	24.37	1.30
7	31.62	31.2	0.67	28.05	28.01	0.07	18.37	18.81	1.18	21.27	22.67	3.19
8	29.31	29.54	0.39	29.12	27.88	2.18	21.1	20.05	2.55	20.64	22.36	4.00
9	28.93	29.88	1.62	25.92	21.25	9.90	20.33	20.87	1.31	24.71	28.12	6.45
10	30.42	29.82	1.00	21.6	22.99	3.11	19.85	19.92	0.18	25.96	29.45	6.30
Mean ± SD	30.59 ± 0.92	31.16 ± 1.04	1.25 ± 0.61	26.76 ± 2.69	26.47 ± 3.44	3.62 ± 2.89	19.27 ± 0.98	18.97 ± 0.98	1.62 ± 0.81	23.20 ± 2.46	23.57 ± 3.49	3.61 ± 2.36

Black boxes indicate which forelimb or hindlimb had higher values. Green indicates decrease and red indicates increase of the values during heelwork walk compared to normal walk. Orange indicates an increase of the values during heelwork walk compared to normal walk but below 3% (normal).

FL, left forelimb; FR, right forelimb; HL, left hindlimb; HR, right hindlimb; IFz, vertical impulse; SI%, symmetry index; SD, standard deviation.

SPD was significantly decreased at the FL and FR (*p* = 0.003 and *p* = 0.001, respectively) and increased at the HL and HR (*p* = 0.001 and *p* = 0.003, respectively).

### 3.3. Vertical force distribution in the paw quadrants

No significant differences of the PFz were found between the normal walk and heelwork walk. IFz was significantly decreased at the CrL quadrant of the FL (*p* = 0.002) and CrL and CrM quadrants of the FR (*p* = 0.038, *p* = 0.009, respectively) and significantly increased in all the quadrants of the hindlimbs except the CrL quadrant of the HL. TPFz was significantly increased at the CdM and CdL quadrant of the FR (*p* = 0.046 and *p* = 0.020, respectively). The area of the CrL quadrant of the FL (*p* = 0.029) was significantly decreased during heelwork walk (Table 6, Figure 2).

**Table 6:** Mean values, standard deviation, and significant differences of the ground reaction forces in the quadrants between normal walking and heelwork walking.

	**PFz (%)**			**IFz (%)**			**TPFz (%)**			**Area (%)**		
	**Mean** ±**SD**		***p*** **-value**	**Mean** ±**SD**		***p*** **-value**	**Mean** ±**SD**		***p*** **-value**	**Mean** ±**SD**		***p*** **-value**
	**Normal walk**	**Heelwork walk**		**Normal walk**	**Heelwork walk**		**Normal walk**	**Heelwork walk**		**Normal walk**	**Heelwork walk**	
FLCaudoLateral	7.28 ± 0.95	7.55 ± 1.41	0.627	6.54 ± 1.36	6.02 ± 1.24	0.386	32.12 ± 6.29	36.01 ± 6.43	0.188	6.94 ± 0.54	7.28 ± 0.71	0.246
FLCaudoMedial	5.57 ± 1.18	5.72 ± 1.23	0.786	4.46 ± 1.17	4.28 ± 0.97	0.716	27.95 ± 5.67	35.17 ± 10.06	0.068	6.41 ± 0.73	6.71 ± 0.67	0.348
FLCranioLateral	8.74 ± 0.98	7.83 ± 1.17	0.075	10.43 ± 1.32	8.13 ± 1.43	0.002^*^	62.39 ± 11.16	60.91 ± 14.30	0.799	6.65 ± 0.63	5.91 ± 0.75	0.029^*^
FLCranioMedial	8.30 ± 0.75	8.14 ± 0.58	0.591	9.17 ± 0.97	8.32 ± 1.10	0.086	69.06 ± 8.82	63.25 ± 13.26	0.266	6.79 ± 0.53	6.55 ± 0.62	0.357
FRCaudoLateral	7.54 ± 1.15	8.20 ± 0.95	0.177	6.67 ± 1.58	6.28 ± 0.93	0.510	30.58 ± 5.94	37.64 ± 6.43	0.020^*^	7.21 ± 0.57	7.40 ± 0.54	0.467
FRCaudoMedial	5.56 ± 0.78	5.60 ± 0.88	0.915	4.77 ± 0.97	3.99 ± 0.66	0.053	29.89 ± 7.34	35.14 ± 7.74	0.046^*^	6.41 ± 0.69	6.40 ± 0.48	0.950
FRCranioLateral	8.62 ± 1.44	7.95 ± 1.24	0.279	10.28 ± 1.76	8.41 ± 1.96	0.038^*^	63.33 ± 10.29	59.34 ± 14.22	0.482	6.55 ± 0.89	6.13 ± 0.79	0.277
FRCranioMedial	8.41 ± 0.88	7.82 ± 1.04	0.188	9.44 ± 1.11	7.78 ± 1.38	0.009^*^	68.53 ± 9.57	62.14 ± 11.87	0.202	6.67 ± 0.80	6.34 ± 0.68	0.337
HLCaudoLateral	4.15 ± 0.77	4.00 ± 0.57	0.629	3.05 ± 0.64	3.64 ± 0.57	0.042^*^	26.47 ± 6.70	24.00 ± 6.53	0.414	5.54 ± 0.60	5.55 ± 0.46	0.954
HLCaudoMedial	4.02 ± 0.73	4.39 ± 0.88	0.318	2.45 ± 0.49	3.45 ± 0.73	0.002^*^	25.03 ± 5.16	21.66 ± 5.95	0.193	5.77 ± 0.45	6.02 ± 0.42	0.225
HLCranioLateral	6.73 ± 1.18	6.42 ± 0.65	0.488	7.89 ± 1.27	8.63 ± 0.94	0.153	57.59 ± 14.19	60.35 ± 9.42	0.615	6.21 ± 0.47	6.17 ± 0.29	0.816
HLCranioMedial	5.36 ± 0.66	5.87 ± 0.60	0.091	5.89 ± 0.86	7.47 ± 1.45	0.010^*^	53.61 ± 17.41	63.12 ± 11.04	0.165	5.77 ± 0.47	5.88 ± 0.35	0.537
HRCaudoLateral	3.99 ± 0.62	4.22 ± 0.98	0.531	2.84 ± 0.49	3.78 ± 0.97	0.017^*^	24.90 ± 4.70	24.08 ± 5.18	0.714	5.51 ± 0.56	5.65 ± 0.75	0.650
HRCaudoLateral	3.99 ± 0.62	4.22 ± 0.98	0.531	2.84 ± 0.49	3.78 ± 0.97	0.017^*^	24.90 ± 4.70	24.08 ± 5.18	0.714	5.51 ± 0.56	5.65 ± 0.75	0.650
HRCaudoMedial	3.82 ± 0.83	4.33 ± 1.29	0.306	2.38 ± 0.60	3.46 ± 1.38	0.042^*^	25.19 ± 5.31	21.78 ± 5.08	0.160	5.57 ± 0.43	5.90 ± 0.51	0.131
HRCranioLateral	6.34 ± 1.12	6.52 ± 1.04	0.716	7.51 ± 1.14	9.20 ± 2.01	0.036^*^	51.32 ± 15.79	58.55 ± 12.82	0.276	5.99 ± 0.60	6.29 ± 0.85	0.374
HRCranioMedial	5.57 ± 0.56	5.44 ± 0.52	0.589	6.24 ± 0.75	7.13 ± 1.07	0.047^*^	56.83 ± 15.40	60.59 ± 14.32	0.579	6.00 ± 0.43	5.82 ± 0.36	0.327

Mean ± SD, mean and standard deviation; PFz%, peak vertical force; IFz%, vertical impulse; TPFz%, time to PFz. Significant differences within the groups are marked with ^*^.

### 3.4. Center of pressure

The COP-radius, COP-area, excursion index, and caudal margin were not significantly different between the heelwork walk and normal walk. The craniocaudal index was significantly decreased at the forelimbs during heelwork walk (FL: *p* = 0.037, FR: *p* = 0.003). The COP-speed was significantly increased at the FL (*p* = 0.012) and FR (*p* = 0.003) during heelwork walk (Table 7, Figure 2).

**Table 7:** Mean values, standard deviation, and significant differences between normal walking and heelwork walking of the center of pressure.

	**COP-area (%)**			**COP-speed (mm/s)**			**COP-radius (%)**		
	**Mean** ±**SD**		***p*** **-value**	**Mean** ±**SD**		***p*** **-value**	**Mean** ±**SD**		***p*** **-value**
	**Normal walk**	**Heelwork walk**		**Normal walk**	**Heelwork walk**		**Normal walk**	**Heelwork walk**	
FL	1.28 ± 0.38	1.32 ± 0.69	0.890	95.61 ± 15.59	129.02 ± 32.66	0.012^*^	0.13 ± 0.02	0.13 ± 0.02	0.427
FR	1.16 ± 0.39	1.26 ± 0.60	0.672	92.68 ± 16.56	126.41 ± 25.27	0.003^*^	0.13 ± 0.02	0.13 ± 0.01	0.320
HL	0.82 ± 0.50	1.07 ± 0.47	0.263	86.67 ± 21.51	102.21 ± 16.31	0.087	0.15 ± 0.03	0.16 ± 0.02	0.383
HR	0.80 ± 0.36	1.12 ± 0.62	0.173	86.39 ± 18.66	101.04 ± 16.04	0.076	0.15 ± 0.02	0.16 ± 0.02	0.269
	**Caudal margin (mm)**			**Craniocaudal index (%)**			**Excursion index (%)**		
	**Mean** ±**SD**		***p*** **-value**	**Mean** ±**SD**		***p*** **-value**	**Mean** ±**SD**		***p*** **-value**
	**Normal walk**	**Heelwork walk**		**Normal walk**	**Heelwork walk**		**Normal walk**	**Heelwork walk**	
FL	32.04 ± 4.05	35.11 ± 3.16	0.076	32.09 ± 7.93	25.06 ± 5.74	0.037^*^	5.12 ± 2.06	7.05 ± 3.55	0.157
FR	32.07 ± 4.15	35.29 ± 2.92	0.062	32.52 ± 7.00	23.10 ± 5.23	0.003^*^	4.21 ± 1.58	5.78 ± 2.18	0.082
HL	31.92 ± 6.12	32.15 ± 2.11	0.909	32.24 ± 7.88	37.12 ± 7.74	0.179	3.14 ± 1.54	3.47 ± 1.62	0.647
HR	32.05 ± 4.60	31.69 ± 2.51	0.834	32.06 ± 8.65	38.10 ± 9.03	0.144	3.30 ± 1.54	3.21 ± 1.81	0.908

FL, left forelimb; FR, right forelimb; HL, left hindlimb; HR, right hindlimb; mean ± SD, mean and standard deviation; COP, center of pressure; Significant differences within the groups are marked with ^*^.

## 4. Discussion

Our hypothesis was that due to the changed head position (lifted and tilted to the right side) during heelwork, higher vGRF will be applied on the left forelimb (due to the tilting to the right) and both hind limbs (due to the lifting) compared to a normal walk This hypothesis was partially confirmed: IFz increased in the hindlimbs. However, the higher loading of the FL did not occur, on the contrary, both forelimbs showed a decreased IFz. Nevertheless, the SI was significantly higher during heelwork than during normal walking, although only slightly above the value that is often used as a cutoff value to differentiate lame from sound dogs (26, 27, 31). Interestingly, when the mean values of PFz and IFz were considered, there were no obvious differences in the values between the right and left forelimb. The SI used nevertheless showed an increasing asymmetry in the load (although not in all animals and within animals also to different extents). This shows the importance of the choice of formula used to calculate the SI, because as described above, some dogs showed higher values on the right and others on the left during heelwork. The study design does not allow further conclusions regarding the differing results between the dogs. However, our results indicate that in further studies, kinematic measurements should be included, which examine the head/neck posture and the back line of the animals. It is likely that the differences in the neck angle described by Harris et al. (14) led to the inconsistent results. The lack of literature on this topic indicates that further studies are needed to develop best practice guidelines for GRFs measurements to obtain an accurate and repeatable results (58).

Due to the lack of scientific studies investigating the influence of head/neck position on the GRFs in dogs, a literature comparison is difficult. However, gait and jump analysis in healthy cats revealed an increase in the PFz of the forelimb to which the head is turned during the measurements by a factor of 1.73 ± 0.53 (59), which was not shown in our study. Furthermore, changes in IFz, stride length, and stance duration of the forelimbs of horses are evident during different positions of the head and neck. A higher degree of collection of the head and neck in horses results in a pronounced shift in impulse toward the hindlimbs (without a similar increase of the PFz) (48). However, a lateral flexion, as seen in dogs during heelwork (11), was not assessed in horses (48). Although the comparisons between different species are difficult due to the different anatomy, we can see some similarities between the biomechanical adaptations when the head and neck position deviates from the natural position. In horses, it has been suggested that a “prolonged stance duration and positive diagonal advanced placement can be indicative of a good balance and self-carriage and interpreted as expression of a greater reliance of the hindlimbs for support” (60). Further studies are needed to investigate if the same applies to dogs.

Regarding the second aim of the study, it could be shown that the particular body position also led to changes in PPD in the forelimbs: the reduction of the forces in the forelimbs was caused by a decrease of IFz in the CrL quadrants of both forelimbs and the CrM quadrant of the FR. Furthermore, a correlation between PPD and COP within the paws could be shown: the reduced loading of the cranial quadrants led to shortened craniocaudal index during heelwork, indicating a changed paw roll over dynamic. This change was accompanied by an increased COP speed in the forelimbs. Although the walking speed of the dogs did not differ between the conditions, a shorter SPD was observed in the front limbs, which together with the shortened craniocaudal index led to a significant increase in COP-speed. In the hindlimbs, on the contrary, the SPD was longer than during normal walk, which, however, did not lead to a change in this parameter due to the unchanged PPD.

In contrast the PPD during heelwork and normal walk was similar in the hindlimbs, leading to no statistically significant changes in COP parameters. This supports our interpretation that craniocaudal index changes are the result adaptations in the pressure distribution within the paw. Similar to our findings, the displacement of COP in human patients with high-arched feet was explained by the differences in pressure distribution (47, 61).

The results of the study can be discussed under different aspects. First, in humans, it is known that the spatial progression of the COP on the craniocaudal foot axis is seen as a function of the articular mobility of the joints in craniocaudal direction in human patients (62), with lower craniocaudal indices indicating reduced joint mobility. To verify whether the changes in COP parameters found here in dogs also lead to changes in joint kinematics, for example in the carpal joint, kinematic measurements would have to be performed in further studies. While the studies on injuries in obedience dogs do not indicate an increased risk for injuries of the pads, it should still be further investigated, if the excessive pressure under the caudal quadrants of the forelimbs contribute to soft tissue injuries, as described in human medicine (63). Furthermore, in human patients, a decreased foot contact area and changes in plantar pressure distribution result in impairments in stability which is explained by a reduction in sensory information (45). While the PCA was not significantly different between heelwork and normal walk, the adaptations in PPD could have a similar effect as described in human medicine, indicating an increased instability.

The caudal margin of the COP and the COP excursion index did not show alterations during heelwork. This is of interest as in lame dogs with elbow dysplasia a larger caudal margin is interpreted as a sign of decreased extension of the limb and consequently an incorrect load takeover of the paw during the beginning of the stance phase (32). If the caudal margin is taken as descriptor of the load takeover, the sound dogs in our study did not displayed a changed load takeover at the beginning of the stance phase during heelwork, but conclusions on changed kinematics of the joints can only be drawn by further studies, as already noted above.

Numerous orthopedic diseases are described in working dogs (6), such as the semitendinosus myopathy (8–10). This disease is of particular interest, since a secondary fibrosis and contracture of the muscle lead to functional impairments and early retirement (9). While the definitive etiology of this disease is still unknown, recurrent microtraumas are discussed as a possible cause (64). The extent to which increased forces acting on the hind legs may contribute to these traumas could be investigated, for example, with parallel measurement of electromyography during work. Furthermore, heelwork could contribute to the progression of existing diseases such as hip dysplasia or worsen the symptoms of lumbosacral stenosis. However, multiple factors play a role here (type of training, training frequency, other required tasks, such as jumping, A-wall, etc.). Nevertheless, the observed changes of vGRF, COP and PPD could contribute to the development of biomechanical pathologies due to altered balance of moments acting across the joint axis, as proposed in human medicine (65–67). However, as mentioned before, simultaneous kinetic and kinematic measurements and electromyography should be performed to achieve a better insight.

One of the limitations of our study was the small sample size. Since this study included only Belgian Malinois and differences exist during gait analysis between different breeds (68–70), we cannot extrapolate our results in other breeds participating in competitive obedience. For example, Della Valle et al. (70) observed that GRFs cannot be compared between breeds with different morphological types. Likewise, Fischer et al. (69) found significant differences in the kinematics of the hindlimbs of Malinois compared to other breeds. This issue complicates the interpretation of the results, since the above-mentioned publication about different neck angles during heelwork included a high percentage of Border Collies (14).

## 5. Conclusion

In conclusion, heelwork leads to specific changes in vGRF, PPD, and COP compared to normal walk, indicating that the COM and consequently the PFz is shifted caudally, the paw rollover dynamics of the front legs display less forces in the cranial quadrants and that the craniocaudal displacement of the COP reacts accordingly. These findings could be also a sign of changed joint kinematics, altered muscle activation and joint reaction forces, which should be investigated in further studies. Such further studies could be used to investigate if these changes contribute to the development of biomechanical pathologies, which are often seen in working dogs.

## Data availability statement

The raw data supporting the conclusions of this article will be made available by the authors, without undue reservation.

## Ethics statement

The animal study was reviewed and approved by Ethics and Animal Welfare Committee of the University of Veterinary Medicine, Vienna. Written informed consent was obtained from the owners for the participation of their animals in this study.

## Author contributions

Conceptualization, methodology, and validation: BB. Formal analysis: AT. Data curation: SK, CL, and DC. Writing—original draft preparation: DC. Writing—review and editing: CL and BB. All authors have read and agreed to the published version of the manuscript.
